# Immunology of Non-Trachomatis Chlamydial Infection

**DOI:** 10.1155/S1064744996000270

**Published:** 1996

**Authors:** M. Poussin, V. Fuentes, J. Orfila

**Affiliations:** Laboratoire de Bactériologie-C.H.U. Nord Place V. Pauchet-80054 AMIENS CEDEX 1 France


Infectious Diseases in Obstetrics and Gynecology 4:122–127 (1996)
© 1996 Wiley-Liss, Inc.

### Immunology of Non-Trachomatis Chlamydial Infection

**M. Poussin, V. Fuentes, and J. Orfila**

*Laboratoire de Bactériologie-C.H.U. Nord Place V. Pauchet-80054 AMIENS CEDEX 1, France*

*Chlamydia* are obligate intracellular parasites with a complexe reproductive cycle. The genus comprises four different species: *C. trachomatis*, *C. pneumoniae*, *C. psittaci* and *C. pecorum*. Most of the work concerning the immune response is found on *C. trachomatis* as it is a major public health problem throughout the world. However the other species are also of interest as *C. psittaci* is one of the most important zoonose in the world and *C. pneumoniae*, recently identified as a separate species, is a common cause of upper and lower respiratory infection worldwide. The seroprevalence of this agent is ranging between 40 and 70%. It has also been suggested that *C. pneumoniae* may play a role in asthma and in coronary disease.

In contrast to *C. trachomatis*, *C. pneumoniae* and *C. psittaci* are able to infect different type of cells such as alveolar macrophages, human monocytes and endothelial cells. This explains the easy access to different organs of the host and the presence of *chlamydia* and their structural components into the circulation. These components can bind to antibodies present in the blood and form immune complexes which maintain inflammatory reactions in the vascular system.

Infections in untreated man and animals with *C. psittaci* sometimes are very severe and may lead to death underlying the importance of the acute phase of these infections. One can notice that the exact physiopathology of this death is not fully understood. Is the endotoxin of *C. psittaci* far more aggressive than the one found in *C. trachomatis?*

For those who recovered the questions of host resistance and immunity arise. As underlined by Storz[33] to understand this problem of natural resistance and acquired immunity one has to consider the nature of these infectious agents as obligate intracellular parasites and their possibility to remain latent.

### IMMUNITY FOLLOWING NATURAL INFECTIONS

Immunity following chlamydiosis is difficult to study but seems to be relative. Stram and Sery reported relapse or reinfection in persons at risk. In chlamydiosis of birds there is a different susceptibility according to species and recovery from an infection leads to immunity.

Stamp, et al.[32] described an increased resistance in ewes after chlamydial abortion. They generally did not abort a second time. Mice developed resistance to reinfection after an experimental infection. Most of the knowledge acquired these last years relied on experiments in mouse models and in guinea pigs models.

### MECHANISM OF NATURAL RESISTANCE

The local reaction to all chlamydial infections is characterised by heavy infiltration with inflammatory cells. During the acute phase polymorphs (PMN) mostly neutrophils predominate within the alveoli. In order to evaluate their role in combating chlamydial infection, Register, et al.[29] examined the effect that *chlamydia* and human PMN have on one another in vitro. They showed that *chlamydiae* exert a chemotactic effect on human PMN in the presence of complement suggesting activation of complement. This phenomenon occurred with and without specific anti *chlamydia* antibodies.

Studies on the uptake and intracellular fate of *chlamydia* was devised to understand the role of this interaction for the outcome of infection. As early as 15 min post inoculation approximately 65% of the PMN-associated EB have been rendered non-infectious probably after fusion of lysosomes with *chlamydia* laden phagosomes and at least half of the infective organisms are rapidly inactivated. It is interesting to note that a small percentage of *C. psittaci* are capable of remaining intact and infec-

Received October 5, 1996
Accepted October 21, 1996

Article tious for a long period of time. They would be capable of infecting macrophages or epithelial cells once they are released from the dying PMN.

PMN infected with *chlamydiae* may be engulfed by macrophages as shown by Newman et al.[24] Those infectious EB may develop in the macrophage and these cells may serve as a vehicle in the dissemination of *C. psittaci* infection.

Thus, as in other infectious diseases, PMN play an ambiguous role both protective in killing EB and deleterious in facilitating the spread of the infectious EB. Here perhaps is one of the physiopathological differences between *C. psittaci* and *C. trachomatis.*

*Chlamydia* can induce interferon in the host. Different experiments have shown an association between induction of interferon in vitro and control of the infection. In vitro, interferon can inhibit inclusion formation.

Another aspect of natural resistance is the control of infection by genetic factors. In mice infected by *C. psittaci* the evolution of the disease is different according to different genotypes. C57BL/6 are strongly resistant showing no symptoms of infection even when massively inoculated whereas BALB/c and DBA/2 are very sensitive. For them death occurs within a few days after inoculation before any installation of acquired immunity. Fuentes[12] tried to study the genetic basis of these different resistances. For that, imbred recombinant C571Bl × BALB/c (C × B) were elaborated.

The first generation hybrids were resistant but a curious phenomenon appeared: In spite of their resistance the number of virulent particles in the spleen of the F1 was as high as that found in the sensitive parent. The dominance is thus linked to the sensitivity. The back-cross mice with the resistant parent developed a bimodal distribution characteristic of a monogenic transmission suggesting 2 possibilities for the localisation of the responsible gene on H 15 and H 30. It is noteworthy to stress that this localisation is different from the one demonstrated for BCG.

### ACQUIRED IMMUNITY

### Antibody Response

*Chlamydia* have a typical cell wall component lipopolysaccharide (LPS) which is highly antigenic and different protein epitopes capable of inducing various kind of antibodies. Animals and humans infected with *C. psittaci* and *C. pneumoniae* show a high level of anti LPS antibodies.

The antibody response of cows and ewes when they are pregnant is characteristic. An initial mild chlamydial antibody response occurs after intramuscular or subcutaneous inoculation. This response is transient and the antibody titer become low or nul before the termination of gestation. The length of the seronegative period before abortion depends on the time interval between inoculation and termination of gestation. If by artificial mechanism the level of antibodies remain high abortion does not occur. It seems that in this special case antibodies play a role in protection.

In other cases the role of antibodies remained ill defined; *Chlamydia*-neutralizing antibodies were demonstrated in pigeons and hyper-immunized roosters[27] but these neutralizing antibodies are seldom found in mammals after recovery of chlamydial infection. In the mouse model developed by Buzoni-Gated passive transfer of monoclonal antibodies protect pregnant mice against abortion and fetal colonisation; placental colonisation was lowered by 5 Lg plaque forming unit. This emphasized the necessity of defining the correct epitope in order to develop protective antibodies. De Sa[9] in the same lab showed the protective ability of a preparation containing native oligomeric MOMP associated with an adjuvant, the LPS of S. typhimuriumRE. In other experimental models when *C. psittaci* is inoculated IV the transfer of antibodies does not protect at all.

Thus even if antibodies may have some neutralising effect on the adhesion phase of infectious EB other mechanisms must be investigated.

An interesting approach to the antibody response was made by Westbay et al.[35] who demonstrated that the LPS of *chlamydia* is a selective factor for the appearance of specific immunoglobulin isotypes. They compared the isotype of antibodies specific for the cysteine-rich outer membrane protein omp2 induced in normal and in LPS hyporesponsive mice. The predominantly IgG2a isotype in LPS hyporesponsive mice is replaced by a high level of IgG1 in LPS responder strains.

This is linked to the structure of the antigen and its natural processing pathway. These results may be of interest when one considers the protective efficacy of the different classes of antibodies.

All chlamydial species have a tendency to cause chronic infections. Persistent *C. psittaci* infections in birds and mammals have been known for years and infections caused by LGV and *C. psittaci* may persist in humans for 10–20 years.

Furthermore, as in the case of *C. pneumoniae*, infected alveolar macrophages or endothelial cells are in close contact with the circulation. By demonstrating the presence of chlamydial LPS-containing immune complexes in diseases associated with possible chronic chlamydial infections like acute myocardial infarction and chronic coronary heart disease[19,21,30] it has been confirmed that chlamydial LPS undoubtedly has an access to the circulation. However, shock-like symptoms have never been described in connection with chlamydial infections but these long-lasting anti-LPS antibodies may play a deleterious role.

Heat shock (or stress) proteins have important functions in cellular metabolism and they aid cells in dealing with environmental stimuli.[20] They are highly conserved and exhibit wide cross-reactivity among eucaryotic cells, parasites and bacteria.[37,38] During acute and chronic infections caused by parasites and bacteria, antibodies to heat shock proteins are produced and these may act as "autoimmune" antibodies and lead to tissue injury. Recent studies have shown that *Chlamydia* possesses at least two heat shock proteins, a 57 kD protein belonging to the 60 hsp family and a 70kD protein belonging to the 70 hsp family.[4,8,22]

Sera from patients with *C. pneumoniae* infection in the USA have been reported to contain antibodies most frequently towards a high molecular weight 98 kDa protein which has been considered *C. pneumoniae* specific,[7] while in chronic infections antibodies frequently recognise 43 kDa and 52 kDa proteins.[28] Two of these proteins, the 98 kDa and 43 kDa proteins, are also recognized by immune complex-bound antibodies in the sera of Finnish patients with chronic coronary heart disease.

Since antibodies are lost after an acute infection[26] and the prevalence rises steadily, it can be estimated that the majority of people have 2 or 3 *C. pneumoniae* infections during their lifetime and evidently primary infection does not protect from reinfections.

The hypothesis concerning the role of heat shock proteins in the pathogenesis of chlamydial infections, first suggested by Morrison, et al.[23] and Bavoil, et al.[2] is the following: chlamydial infections are usually limited anatomically and temporally by potent cell mediated and humoral immune responses. After the primary infection, hypersensitivity reactions with tissue injury can occur upon re-exposure to the organism, such as in trachoma. During this phase organisms are rarely isolated, but inflammatory symptoms are strong. Recent data on the role of mycobacterial stress proteins and auto-reactive T-lymphocytes suggest autoimmune mechanisms in the pathogenesis of chronic mycobacterial disease. These mechanisms might also play a role in the immunopathologic sequelae of chronic chlamydial infections.

### Cell Mediated Immune Response

T cell mediated immunity is reported to be important in the defense against intracellular pathogens. Drobyshevskaya, et al.[10] as early as 1962, studying normal and immunized mice inoculated intranasaly by CE, demonstrated a marked quantitative difference between the two groups in the dynamics of multiplication of the *chlamydia* with a lower titer in the immunized group. In contrast to control mice chlamydial multiplication in the macrophages of vaccinated mice was limited and a larger proportion of infected cells remained viable. Macrophages and large round cells appeared in the lungs of immunized mice concentrated around blood vessels forming dense cellular cuffs. Lymphocytes and histiocytic cells with mitotic figures were present in these perivascular aggregates of immunized mice.

This early work stresses the importance of macrophages and lymphocytes. Many different experiments were subsequently performed in order to analyse the different organisms involved.

*Chlamydia psittaci* (Cp) avian strain Loth (Loth) has been shown to be very pathogenic for different strains of mice: DBA/2, C3H/He and BALB/c.[11] For these mice, the inoculation of Loth strain results in the death of animals in 3 to 10 days. The same effect is observed with the 6BC strain of Cp. With this strain, the intraperitoneal inoculation of a single elementary body is lethal for sensitive mice. Byrne[6] showed that BALB/c mice can be protected with a sublethal intramuscular inoculation with the 6BC strain.

However immunity can be demonstrated using another less virulent strain the ovine abortion strain A/22.[22] BALB/c mice inoculated with this A/22 strain remain perfectly healthy and are protected when reinoculated with the lethal Loth strain. This model can be used to elucidate the mechanisms involved in this crossed immunity.

Survival of Loth-inoculated mice was considered as definitive after 3 weeks post-Loth inoculation. For transfers experiments, mononuclear spleen cells were first purified by Ficoll/Paque density Gradient and further selected using CD4 or CD8 enrichment columns. Production of cytokines and inducible NO synthase transcripts were studied using RT-PCR. Secretion of specific IgG antibodies by splenic cells was investigated.

Survival-based considerations clearly demonstrate that the A/22-induced protection is Cp-specific (protection against Loth cannot be induced by *Chlamydia trachomatis* nor *Chlamydia pneumoniae*, and A/22-treated mice are not protected against the other intracellular bacteria studied: *Listeria monocytogenes*). Mice are protected against Loth for more than one year after A/22 immunisation.

Transfer experiments using splenic mononuclear cells from protected and unprotected mice imply that cells responsible for such a protection are $CD4^+$ T lymphocytes, and that transfer of serum from immune mice does not confer any protection to recipient mice.

Among $CD4^+$ T lymphocytes, two subpopulations exist, Th1 and Th2 cells, which are defined by their cytokines synthesis patterns. Cytokine mRNA synthesis in splenic mononuclear cells, as well as the isotype of excreted Cp-specific IgG, show that protected mice exhibit a Th1-type response. This kind of immune response is established by a preferential production of IFN-$\gamma$, TNF$\alpha$, TNF$\beta$ and by a typical predominance of $IgG_{2a}$ among specific IgG antibodies. It is also characterised by cellular effector mechanisms.

One of the effector mechanisms involved seems to be the induced nitric oxide (NO) production in macrophages during healing of the mice. Production of messenger RNA for inducible NO synthase could be detected in the peritoneum of A/22-protected BALB/c mice, as well as in naturally resistant C57BL/6 mice, after intraperitoneal inoculation with Loth strain.

This data indicate that Th1-$CD4^+$ T lymphocytes and activated macrophages are implicated in immunity against Cp. Other cellular mechanisms of bacterial clearance (NK cells or $CD8^+$ T lymphocytes) are probably also involved. This requires further studies. Nevertheless, this model of induced immunoprotection will be a tool to evaluate vaccine preparations against *Chlamydia* sp. It will also be useful to increase our knowledge of the immune response necessary to cure an acute chlamydial infection.

T-cell mediated immunity against *C. pneumoniae* has thus far been studied in persons who have acquired a laboratory infection caused by *C. pneumoniae* strain Kajaani 6.[34] A definite antigen-specific lymphocyte proliferation response, which started to increase at 3 weeks and had a peak value at 8–9 weeks after the onset of symptoms, was recorded. T-cell responses to *C. pneumoniae* antigens are significantly stronger in healthy, seropositive individuals than in seronegative persons. In contrast to healthy individuals in whom the T-cell reactivity is specific to *C. pneumoniae* antigens, we have recently observed that patients with severe coronary heart disease elicit a T-cell response that is cross-reactive with different chlamydial species and which might be directed against conserved protein epitopes in MOMP or even against chlamydial heat shock proteins shared by different chlamydial species. Moreover, high antibody titers to *C. pneumoniae* in patients with coronary heart disease were often associated with a decreased T-cell reactivity against *C. pneumoniae*. These preliminary results suggest that T-cell immunity might be important in the protection against *C. pneumoniae*, and that in chronic infections or in reinfections T-cells may also be responsible for the immunopathological processes associated with the disease.

It has been shown that *C. pneumoniae* is capable of inducing IL1 and TNF production in human monocytes.[13] We have also shown that in acute *C. pneumoniae* pneumonia, triglyceride levels are significantly higher and HDL levels, particularly HDL2, significantly lower than in either viral or pneumococcal pneumonias. It can be speculated that in chronic *C. pneumoniae* infections the continuous production of TNF might lead to a serum lipid pattern similar to that which is considered as a risk factor for coronary heart disease.

Recent data[17] on reinfection in mice indicate that bacterial cultures are positive only for a couple of days after the rechallenge. However, the inflammatory changes in the lungs are as strong and long-lasting and develop earlier than the changes seen in primary infection. When convalescent or hyperimmune serum is given intraperitoneally prior to the intranasal challenge, *Chlamydia* cultures stay negative, but the lung histology shows acute pneumonia with polymorphonuclear leukocytes.

The results from animal studies suggest that antibodies, i.e. humoral immunity, may contribute to protection and that the strong inflammatory response seen in the lungs after the rechallenge of mice with *C. pneumoniae* may be due to the immunodestructive action of T-cell mediated immunity.

### BIBLIOGRAPHY

1. Ata FA: Factors influencing clearance of chlamydial agents in sheep. Ph. D. Thesis, Fort Collins, Colorado State University, 1970.
2. Bavoil P, Stephens RS, Falkow S: A soluble 60 kDa antigen of *Chlamydia spp.* is homologue of Escherichia coli GroE1. Molec Microbiol, 4:461–469, 1990.
3. Beatty RP, Stephens RS: CD8+ T lymphocyte-mediated lysis of *Chlamydia*-infected L cells using an endogenous antigen pathway. J Immunol 153:4588–4595, 1994.
4. Birkelund S, Lundemose AG, Christiansen G: The 75 kDa cytoplasmatioc *Chlamydia trachomatis* L"2 polypeptide is a DNA-like protein. Infect Immun 58:2098–2104, 1990.
5. Byrne GI, Faubion CL: Lymphokine-mediated microbistatic mechanisms restrict *Chlamydia psittaci* growth in macrophages. J Immunol 128:469–474, 1982.
6. Byrne G, Padilla M, Lacy D, Paulnock D, Guo XL: Mouse model for protective immunity to *Chlamydia*. 7th International Symposium on Human Chlamydial Infection, Harrisson Hot Springs (British Columbia), 1990.
7. Campbell LA, Kuo CC, Wang SP, Grayston JT: Serological response to *Chlamydia pneumoniae* infection. J Clin Microbiol 28:1261–1264, 1990.
8. Cerrone MC, Ma JJ, Stephens RS: Cloning and sequence of the gene for heat shock protein 60 from *Chlamydia trachomatis* and immunological reactivity of the protein. Infect Immun 59:79–90, 1991.
9. De Sa C, Bernard D, Salinas J, Souriau A, Rodolakis A: Protection against *Chlamydia psittaci* infection in a mouse model with a vaccine containing native oligomeric Major Outer Membrane Protein. In: "Proceedings third meeting of the European Society for Chlamydia research" Stary (ed). Vienne (Autriche) 11–14 Sept, 1996.
10. Drobyshevskaya AI, Pigarevsky VE, Smorodintsev AA: Activity of phagocytic factors in experimental infection of white mice with mouse pneumonia and meningopneumonia viruses. Acta Virol 6:458–470, 1962.
11. Fuentes V, Orfila J: Genetic control of natural resistance to *Chlamydia psittaci* Loth strain in Mice. 7th International Symposium on Human Chlamydial Infection, Harrisson Hot Springs (British Columbia), 1990.
12. Fuentes V, Puy H, Lefebvre JF, Orfila J: Specific immunity to *Chlamydia psittaci* in the mouse: crossed immunity between ovine abortion and avian strains. Microbios Letters 40:55–62, 1989.
13. Howard LV: Neutralization of *Chlamydia trachomatis* in cell culture. Infect Immun 11:698–703, 1975.
14. Igietseme JU, Magee DM, Williams DM, Rank RG: Role of CD8+ T cells in antichlamydial immunity defined by *Chlamydia*-specific T-lymphocytes clones. Infect Immun 62:5195–5197, 1994.
15. Karimi ST, Schloemer RH, Wilde CE III: Accumulation of chlamydial liposaccharide antigen in the plasma membranes of infected cells. Infect Immun 57:1780–1785, 1989.
16. Kunimoto DY, Nordan RP, Strober W: IL-6 is a potent cofactor of IL-1 in IgM synthesis and of IL-5 in IgA synthesis. J Immunol 143:2230–2235, 1989.
17. Laitinen K, Laurila A, Leinonen M, Saikku P: Experimental *Chlamydia pneumoniae* infection in mice: effect of reinfection and passive protection by immune serum. In: Proceedings of the 8th International Symposium on Human Chlamydial Infections, Chantilly 19–24 June 1994, Orfila J, et al (Eds) Esculapio, Bologna, 545–548.
18. Lammert JL: Cytotoxic cells induced after *Chlamydia psittaci* infection in mice. Infect Immunol 35:1011–1017, 1982.
19. Leinonen M, Linnanmaki E, Mattila K, Nieminen MS, Leirisalo-Repo M, Valtonen V, Saikku P: Circulating immune complexes containing Chlamydial lypopolisaccharide in acute myocardial infarction. Microbiol Pathogen 9:67–73, 1990.
20. Lindquist S, Craig EA: The heat shock proteins. Ann Rev Genet 22:631–677, 1988.
21. Linnanmarki E, Leinonen M, Ekman MR, Mattila K, Nieminen MS, Valtonen V, Saikku P: *Chlamydia pneumoniae* specific circulating immune complexes in chronic heart disease. Circulation 87:1130–1134, 1993.
22. Menozzi FD, Menozzi-Dejaiffe C, Nano FE: Molecular cloning of a gene encoding a *Chlamydia psittaci* 57 kDa protein that shares antigenic determinants with ca. 60 kDa proteins present in many gram-negative bacteria. FEBS Letters 58:59–64, 1989.
23. Morrison RP: Chlamydial 57 kDa stress response protein is deleterious immune target: In: Microbial determinants of virulence and host response. Ayuoub EM et al. (eds), American Society of Microbiology, Washington DC, 1990, 243–250.
24. Newman S, Heuson C, Henson PM: J Exp Med 156:430–442, 1982.
25. Paquirigan AM, Byrne GI, Becht S, Carlin JM: Cytokine-mediated indoleamine 2,3-dioxygenase induction in response to *Chlamydia* infection in human macrophage culture. Infect Immun 62:1131–1136, 1994.
26. Patnode D, Wang SP, Grayston JT: Persistance of *Chlamydia pneumoniae*, strain TWAR, microimmunofluorescence antibody. In: Proceedings of the 7th International Symposium on Human Chlamydial Infections, Bowie WR et al (eds). 406–409, 1990.
27. Piraino FF: The occurrence of psittacosis virus complement-fixing (CF), noncomplement-fixing (NCF) and neutralizing (N) antibodies in domestic pigeons. J Immunol 95:1107–1110, 1965.

28. Puolakkainen M, Kuo CC, Shor A, Wang SP, Grayston JT, Campbell LA: Serological response to *Chlamydia pneumoniae* in adults with coronary arterial fatty streaks and fibrolipid plaques. J Clin Microbiol 31:2212–2214, 1993.
29. Register KB, Morgan PA, Wyrick PB: Interaction between *Chlamydia spp.* and human polymorphonuclear leukocytes in vitro. Infect Immun 52:664–670, 1986.
30. Saikku P, Leinonen M, Tenkanen L, Ekman MR, Linnanmarri E, Manninen V, Manttari M, Frick MM, Huttunen JK: Chronic *Chlamydia pneumoniae* infection as a risk factor for coronary heart disease in the Helsinki heart study. Ann Int Med 116:273–278, 1992.
31. Salinas J, Sanchez J, Buendia AJ, Souriau A, Rodolakis A, Bernabe A, Cuello F: The LPS localization might explain the lack of protection of LPS-specific antibodies in abortion-causing *Chlamydia psittaci* infections. Res Microbiol 145:611–620, 1984.
32. Stamp JT, McEwen AD, Watt JAA, Nisbet DJ: Enzootic abortion in ewes. I. Transmission of the disease. Vet Rec 62:251–254, 1950.
33. Storz J, McKercher DG: Antibody response before and after abortion in psittacosis (*Chlamydia*) infections of pregnant cows. Cornell Vet, 60:192–203, 1970.
34. Surcel HM, Syrjala H, Leinonen M, Saikku P, Herva E: Cell mediated immunity to *Chlamydia pneumoniae* measured as lymphocyte blast transformation in vitro. Infect Immunol 61:2196–2199, 1993.
35. Westbay TD, Dascher CC, Hsia RC, Zauderer M, Bavoil PM: Infect Immun 63:1391–1393, 1995.
36. Yang Z-P, Kuo CC, Grayston JT: A mouse model of *Chlamydia pneumoniae* strain TWAR pneumonitis. Inf Immunol 61:2037–2040, 1993.
37. Young DB, Ivanyi J, Cox JH, Lamb JR: The 65 kDa antigen of mycobacteria a common bacterial protein. Immunol Today 8:215–219, 1987.
38. Young D, Lathigra R, Hendrix R, Sweetser R, Young RA: Stress proteins are immune targets in leprosy and tuberculosis. Proc Natl Acad Sci 85:4267–4270, 1988.
